# Application and performance of deep learning models for the automated diagnosis of cervical central spinal stenosis on MRI: a systematic review

**DOI:** 10.1016/j.bas.2025.105902

**Published:** 2025-12-18

**Authors:** Vasileios Mougios, Robin Peretzke, Alexandra Ertl, Martin Dugas, Klaus Maier Hein, Peter Neher, Sebastian Ille, Sandro Krieg, Pavlina Lenga

**Affiliations:** a Department of Neurosurgery, Heidelberg University Hospital, Heidelberg, Germany; b German Cancer Research Center, Division of Medical Image Computing, Heidelberg, Germany; c Pattern Analysis and Learning Group, Department of Radiation Oncology, Heidelberg University Hospital, Heidelberg, Germany; d Institute of Medical Informatics, Heidelberg University Hospital, Heidelberg, Germany

**Keywords:** AI, Deep-learning, Convolutional neural network, MRI, Cervical central canal stenosis

## Abstract

**Introduction:**

Cervical central spinal stenosis (CCSS) is a leading cause of adult spinal cord dysfunction. Magnetic resonance imaging (MRI) is the reference standard, but reporting is time-consuming and subject to inter-observer variability. Artificial intelligence (AI)—especially deep-learning—may enable automated, consistent assessment.

**Research question:**

Evaluation of performance metrics of AI models for diagnosing CCSS.

**Material and methods:**

Following PRISMA 2020, we searched PubMed, Cochrane, Embase, IEEE Xplore, and Web of Science (2015–July 2025) for studies training and evaluating AI models using MRI to diagnose or grade CCSS. We excluded studies limited to foraminal stenosis, non-MRI modalities, thoracic/lumbar levels, segmentation-only or image-enhancement tools without diagnostic output, and studies focused solely on non-stenotic cervical pathologies. Data were extracted on MRI protocol, model type, data splits and external validation, stenosis classification, and diagnostic performance.

**Results:**

Ten studies (2019–2025) met inclusion criteria, predominantly single-centre and retrospective. Most models used T2-weighted axial and/or sagittal MRI; CNNs (e.g., ResNet-50, EfficientNet) and Transformer-based architectures were common. Sensitivities ranged roughly 0.67–1.00 and specificities 0.42–0.97 across models, with many reporting AUCs ≥0.90 and accuracies ≥0.85. Only one study reported true external test performance. Reporting of confidence intervals, processing time, and explainability (e.g., Grad-CAM) was inconsistent.

**Discussion and conclusion:**

Deep-learning shows promising diagnostic performance for automated CCSS assessment on MRI and could reduce variability and reporting time. However, generalisability remains uncertain due to small, retrospective, largely single-centre cohorts and scarce external validation. Standardized reporting (e.g., CLAIM) and prospective, multi-centre validation is needed before routine clinical deployment.

## Highlights

• Systematic review of application of AI deep learning on MRI for the diagnosis of cervical central spinal stenosis.
• Inclusion of CNN and Transformer-based models.
• Most models achieved high diagnostic performance (AUC ≥0.90; accuracy ≥0.85).
• External validation was rare; limited comparability due to reporting heterogeneity.
• Standardized reporting and multicentre prospective validation are essential.

## 1 Introduction

Cervical central spinal stenosis (CCSS) is a common degenerative condition resulting in narrowing of the spinal canal of the cervical spine. Stenosis of the cervical canal can present with a multitude of symptoms ranging from non-specific symptoms like axial neck pain to radicular symptoms to myelopathy and disturbances of autonomic function(Jiang et al., 2024a, 2024b; Sharma et al., 2025; Meyer et al., 2008)–(Jiang et al., 2024a, 2024b; Sharma et al., 2025; Meyer et al., 2008). It is a leading cause of spinal cord dysfunction in adults, with prevalence increasing due to aging populations(Smith et al., 2021). Moreover, the duration of symptoms substantially precedes the referral to further assessment and treatment with an estimate of >1 year before final diagnosis as reported in the meta-analysis from Malone et al. (2025) As a result, early diagnosis is essential, as timely surgical decompression might be beneficial even in patients with radicular pain and mild cervical myelopathy in order to prevent further neurological deterioration through long-standing spinal cord trauma(Fasinella et al., 2025; Connelly et al., 2024).

Magnetic resonance imaging (MRI) remains the reference standard for CCSS diagnosis because of its superior soft-tissue and spinal cord resolution and ability to assess cord compression. Furthermore, other techniques other than the standard and widely-used T1/T2 sequences can be employed to delineate structural changes of the spinal cord itself(Nouri et al., 2016; He et al., 2022). However, MRI interpretation can be time-consuming and impose a substantial burden on personnel, particularly in high-volume medical centres, while being subject to interobserver variability and reporting discrepancies despite the use of standardized grading scales(Fahmy et al., 2023; Stafira et al., 2003).

Artificial intelligence (AI), particularly deep learning methods such as convolutional neural networks (CNN), has proved to be a promising means of automating detection of spinal degenerative changes and has been extensively applied in the lumbar spine(Zhang et al., 2023; Wang et al., 2024; Ghobrial and Roth, 2025; Liawrungrueang et al., 2025a; Lin et al., 2024) (Zhang et al., 2023; Wang et al., 2024; Ghobrial and Roth, 2025; Liawrungrueang et al., 2025a; Lin et al., 2024). However, the use of AI in automated detection, grading and assessment of CCSS has been less investigated.

The aim of this systematic review is to evaluate the application of AI deep learning models' diagnostic performance for the automated detection of CCSS on MRI.

## 2 Methods

### 2.1 Literature search

A systematic bibliographic search of the available literature was performed using the 2020 PRISMA Guidelines(Page et al., 2021). As the topic of this review contains both medical and technical elements a search was performed through June and July 2025 in PubMed, Cochrane, EMBASE, IEEE Xplore and Web of Science. The year of publication was restricted between 2015 and 2025. The terms used for the search in the databases can be found in the Supplementary Material.

Studies were included if they directly addressed the use of AI models in combination with MRI images to diagnose and/or grade and/or assess the severity exclusively of cervical canal stenosis.

Studies were excluded if they met any of the following criteria: (1) exclusively foraminal stenosis, (2) other imaging modalities (X-ray, CT), (3) other spine levels (thoracic, lumbar), (4) publication before January 1, 2015, (5) AI models with endpoints other than automated diagnosis/classification of cervical canal stenosis (e.g. image enhancement, automated segmentation) or segmentation-only tools without diagnostic outputs, (6) AI models utilized for the diagnosis of other cervical diseases (myelopathy not attributed to CCSS, osteophytes, disc changes, OPLL, trauma etc.)

### 2.2 Data extraction

The data extracted from the final articles collected for review were the following: (1) year of publication, (2) MRI tesla field strength (T), sequence and imaging plane, (3) pre-processing and image augmentation steps, (4) number of images used for training, validation and testing, (5) presence of external validation, (6) AI model type, (7) use of segmentation tool, (8) stenosis grading classification and (9) performance metrics of the respective AI model (recall rate, F1-score, specificity, sensitivity, AUC-score, PPV, NPV, accuracy, k-value of AI model, process time). Furthermore, the reported confidence intervals for specificity, sensitivity and AUC-scores were extracted.

Only performance metrics directly reported by the authors were extracted. Secondary calculations of additional metrics from available study data were not performed, as this was deemed potentially imprecise. This approach was chosen since performance metrics derived indirectly from incomplete or heterogeneous data might lead to bias.

## 3 Results

### 3.1 Article selection

**Fig. 1:**
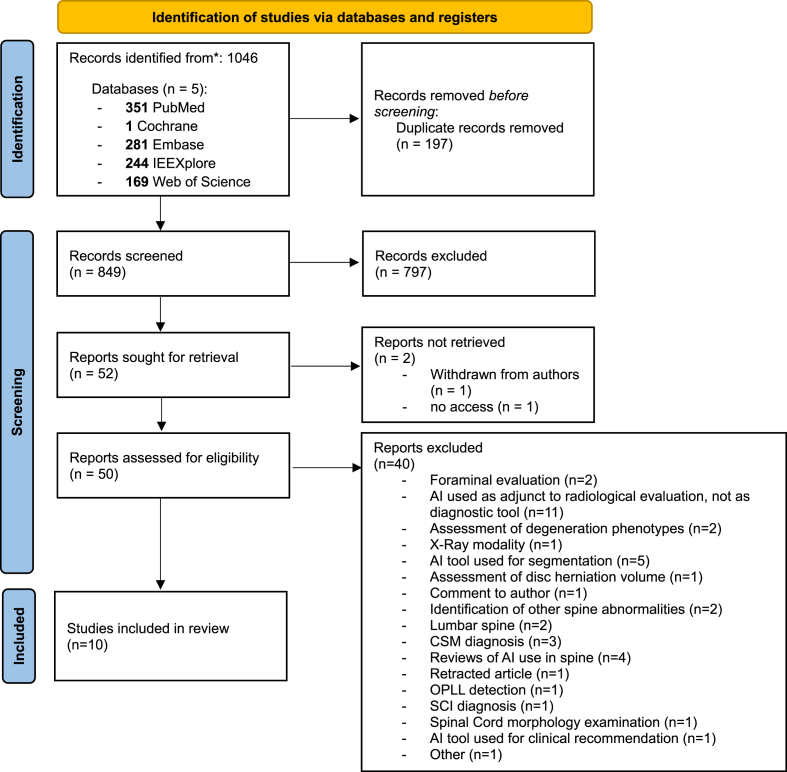
PRISMA 2020 flow diagram.

The initial database search identified 1046 records across five databases: PubMed (n = 351), Cochrane Library (n = 1), Embase (n = 281), IEEE Xplore (n = 244), and Web of Science (n = 169). After removal of 197 duplicates, 849 records were screened according to title and abstract. Of these, 797 were excluded as irrelevant. Another 2 articles were excluded, as 1 of them was withdrawn from the authors and 1 was not retrieved. The remaining 50 full-text articles were assessed for eligibility with 40 excluded (foraminal stenosis evaluation only, AI model used for segmentation, image quality improvement, assessment of other pathologies of the cervical spine, other radiographic modalities other than MRI used, AI use in lumbar spine, diagnosis and evaluation of myelopathy only or spinal cord injury). 10 studies met all eligibility criteria and were included in the final review. The PRISMA flow diagram is presented in Fig. 1.

### 3.2 Study Characteristics

The 10 included studies were published between 2019 and 2025, with the majority conducted in single-centre and 2 studies in multicentre settings. 8 of them were of retrospective design. The majority of the studies were conducted in South Korea and China.

### 3.3 MRI sequence, imaging plane and tesla field strength (T)

MRI field strengths varied, with most studies utilizing both 1.5T and 3.0T scanners. T2-weighted sequences (axial and/or sagittal) were the most common inputs; several studies incorporated gradient echo or multi-sequence inputs for model training. Furthermore, the majority of the AI models were trained using axial images of cervical spine MRI images apart from Hohenhaus et al. (2024) who used a 3D imaging plane. The relevant data can be found in Table 1.

**Table 1:** MRI sequence, Tesla-field strength and imaging plane used for training of AI models. (NR = not reported).

Author	MRI sequence	MRI Tesla Strength	Imaging plane for training
Merali et al. (Merali et al., 2021)	T2-weighted non-fat-saturated	NR	axial
Payne et al. (Payne et al., 2024)	Gradient Echo (GRE)	1.5 and 3T	axial
Lee et al. (Lee et al., 2025a)	T2-weighted spin-echo, T2-weighted gradient-echo	1.5 and 3T	axial; sagittal
Zhang et al. (Zhang et al., 2024)	GRE, T2∗MERGE, T2 FRFSE	1.5 and 3T	axial
Rhee et al. (Rhee et al., 2025)	T2-weighted	NR	sagittal
Wang et al. (Wang et al., 2025)	T2 spin-echo	3T	sagittal
Hopkins et al. (Hopkins et al., 2019)	Multi-echo gradient-echo sequence	3T	axial
Lee A. et al. (Lee et al., 2025b)	T2-weighted spin-echo, T2-weighted gradient-echo	1.5 and 3T	axial
Abuhayi et al. (Abuhayi et al., 2024)	NR	NR	axial; sagittal
Hohenhaus et al. (Hohenhaus et al., 2024)	3D T2-weighted SPACE	3T	3D multiplanar (SPACE)

### 3.4 MRI parameters, pre-processing and data augmentation steps

Regarding the MRI parameters, pre-processing and augmentation steps there exists a substantial heterogeneity among articles. The data are presented in Table 2.

**Table 2:** MRI parameters, pre-processing and data augmentation. (NR = not reported).

Author	MRI parameters	Pre-processing	Data augmentation
Merali et al. (Merali et al., 2021)	Slice thickness median (range) 3 (2–5) mm, Voxel size median (range) 0.3516 (0.2539–0.7813) mm	Anonymization using DicomCleaner. Down-sampling of each image to 299 × 299 JPEG format. Normalization of pixel values between 0 and 1. Storage of each image in a 299 × 299 × 3 array. Utilization of RGB grayscale format	NR
Payne et al. (Payne et al., 2024)	TR: 538–761 ms, TE: 13 ms, Flip angle: 30°, FOV: 165–180 mm, number of excitations: 1–3, section thickness: 3 mm, section gap: 0 mm, matrix size: 320–384 128–192, slices: 34–64, sequence acquisition time: 2.5–4.0 min	Anonymization, Conversion to NIfTI format	NR
Lee et al. (Lee et al., 2025a)	**Axial** TR: 533–1200 ms, TE: 14–20 ms, section thickness: 3 mm, FOV: 160–180 mm **Sagittal** TR: 2000–5200 ms, TE: 80–105 ms, section thickness: 3 mm, FOV: 200–240 mm	Anonymization, DICOM format	NR
Zhang et al. (Zhang et al., 2024)	FOV: 128–256 mm, Slice thickness: 4 mm, TR: 400–608 ms, TE: 5.5–17 ms, Flip angle: 20°	Anonymization, DICOM format preservation	NR
Rhee et al. (Rhee et al., 2025)	mean ± SD TE: 112 ± 15 ms, mean ± SD TR: 3360 ± 710 ms, matrix size: mostly 512 × 512, slice thickness: 3 mm	Extraction from DICOM files using Pydicom (version 2.4.4), anonymization. Preprocessing using OpenCV-Python library. Grayscale-RGB designation and merging; Cropped and resized to 224 × 112 × 3	Random rotation from −10 to +10°, random scaling from −10 to +10 %.
Wang et al. (Wang et al., 2025)	NR	Selection of mid-sagittal T2-weighted images using MicroDicom 2022.1; Conversion to JPG format	Random horizontal flipping; random vertical flipping; random rotation (−10°–10°); contrast enhancement (1–1.5 × ); brightness variation (0.7–1.3 × ); normalization
Hopkins et al. (Hopkins et al., 2019)	TR = 300 ms, TE = 18 ms, Flip angle = 30°, FOV = 180 × 180, Matrix size = 384 × 384, In-plane resolution = 0.47 × 0.7 mm2, Slice thickness = 4 mm, averages = 2	NR	NR
Lee A. et al. (Lee et al., 2025b)	NR	image resizing, pixel intensity distribution normalization, batch normalization, layer normalization	NR
Abuhayi et al. (Abuhayi et al., 2024)	NR	Image resize to 224 × 224, one-hot encoding	Random rotations, flips, zoom (using Keras ImageDataGenerator)
Hohenhaus et al. (Hohenhaus et al., 2024)	voxel size 0.6 mm × 0.6 mm × 1.0 mm, TR 1500 ms, TE 134 ms, Flip angle 105°, GRAPPA PAT: 3, acquisition time 3:53 min	NR	NR

### 3.5 Number of images used for training, validation and testing, train:validation:test split, presence of external validation

The number of images used for training, validation and testing for AI models presents also a substantial heterogeneity across the studies found. Only Lee et al. (2025a) used an external dataset of images for testing. The results are presented in Table 3. Sample sizes varied substantially, from slightly fewer than 1000 to over 9000 images in Merali et al. (2021) in combined training, validation, and test sets.

**Table 3:** Number of images, train:validation:test split, presence external validation. (NR = not reported).

Author	No. Images	Train:Validation:Test Split	External validation
Merali et al. (Merali et al., 2021)	9579 (calculated)	75 %(Training and Validation):25 %(Test)	No
Payne et al. (Payne et al., 2024)	2811 (calculated)	60 %:20 %:20 %	No
Lee et al. (Lee et al., 2025a)	NR	90 %(Training):10 %(Test)	Yes
Zhang et al. (Zhang et al., 2024)	3688	5-fold cross-validation	No
Rhee et al. (Rhee et al., 2025)	NR	90 % (3-fold cross-validation):10 %	No
Wang et al. (Wang et al., 2025)	954	80 %:20 %, 5-fold cross-validation	No
Hopkins et al. (Hopkins et al., 2019)	NR	cross-validation using random partitions	No
Lee A. et al. (Lee et al., 2025b)	2955	NR	No
Abuhayi et al. (Abuhayi et al., 2024)	NR	60 %:20 %:20 %	No
Hohenhaus et al. (Hohenhaus et al., 2024)	1010	125(Training):5(Validation)	No

### 3.6 AI model architecture, presence of segmentation and stenosis classification, use of Grad-CAM, threshold

AI model architectures varied widely. Convolutional neural networks (CNNs) were the most common, including ResNet-based architectures, Faster R-CNN, and attention-based models. One study employed a Vision Transformer (ViT) fine-tuned on ImageNet. Most models used already established radiological classifications, such as the Kang classification MRI grading system(Kang et al., 2011), or modifications of it. Substantial heterogeneity exists also in the utilization of segmentation tools. External testing was conducted only in 1 study(Lee et al., 2025a), while the use of Grad-CAM was performed in 2 studies (Merali et al., 2021; Rhee et al., 2025). The relevant information can be found in Table 4. A summary of the Kang(Kang et al., 2011) and Muhle(Muhle et al.) classifications is presented in Table 5.

**Table 4:** AI model architectures, presence of segmentation, stenosis classification, Grad-CAM use, threshold. (NR = not reported).

Author	AI model	Segmentation	Stenosis classification	Threshold	Gad-CAM
Merali et al. (Merali et al., 2021)	ResNet-50, transfer-leaning using ImageNet	No	binary (compressed/non-compressed)	0.5 (dichotomized)	Yes
Payne et al. (Payne et al., 2024)	ViT model pretrained on ImageNet was fine-tuned by using PyTorch; ResNet-50; DenseNet121	Yes (ITK-SNAP)	Kang classification	NR	No
Lee et al. (Lee et al., 2025a)	ResNet-50 utilizing an attention-based model	Yes (Darwin V7)	Muhle classification	0.5 (dichotomized)	No
Zhang et al. (Zhang et al., 2024)	Faster R-CNN, consisting of a ROI detection module and cascade classification prediction	Yes (unnamed open-source)	quaternary (0–3)	0.5 (dichotomized)	No
Rhee et al. (Rhee et al., 2025)	ResNet-50, VGG16, MobileNetV3, EfficientNetV2, Ensemble model. Transfer learning using ImageNet-pretrained CNN models	No	Kang classification	0.5 (dichotomized)	Yes
Wang et al. (Wang et al., 2025)	EfficientNet-B5, weights pre-trained on ImageNet	No	Park-modified Kang classification	NR	No
Hopkins et al. (Hopkins et al., 2019)	Deep neural network; trained using Keras open source Python package	No	Kang, Nagata and Chang grading systems. MRI radiomics (sagittal canal width, vertebral body height to vertebral disk height ratio, and the C5 vertebral body sagittal width)	NR	No
Lee A. et al. (Lee et al., 2025b)	ResNet-50, pre-trained with COCO, with transformer	Yes (Darwin V7)	Muhle classification	dichotomized	No
Abuhayi et al. (Abuhayi et al., 2024)	Inv-AlxVGGNets: concatenated AlexNet-inspired branch with Involution layers + VGG-inspired branch with residual layers	No	NR	NR	No
Hohenhaus et al. (Hohenhaus et al., 2024)	Patchwork CNN based on TensorFlow (>2.0), Keras and NiBabel	Yes (in-house pipeline, NORA)	Kang classification, adapted Maximal Canal Compromise (aMCC), adapted Spinal Cord Occupation Ratio (aSCOR)	aMCC: 1.18 (“no” vs “relative”), 1.54 (“relative” vs “absolute”); aSCOR: 36.9 % (“no” vs “relative”), 49.3 % (“relative” vs “absolute”)	No

**Table 5:** Cervical stenosis classification adapted from Kang et al. (2011) and Muhle et al. (Muhle et al.)

Stenosis Classification	Grade 0	Grade 1	Grade 2	Grade 3	Grade 4
Kang et al. (Kang et al., 2011)	Normal (no stenosis)	>50 % subarachnoid space obliteration, no spinal cord deformity	subarachnoid space obliteration, spinal cord compression, no spinal cord signal change	Spinal cord compression with increased spinal cord signal intensity	
Muhle et al. (Muhle et al.)	Normal disc height, no degenerative signs	Degenerative signs without no osteophytes; anterior cord compression possible	Spondylosis and/or osteochondrosis; anterior cord compression possible	Spondylosis and/or osteochondrosis with bridging osteophytes; anteroposterior cord compression possible	Cervical spondylotic myelopathy; anteroposterior cord compression possible

### 3.7 Performance metrics

**Fig. 2:**
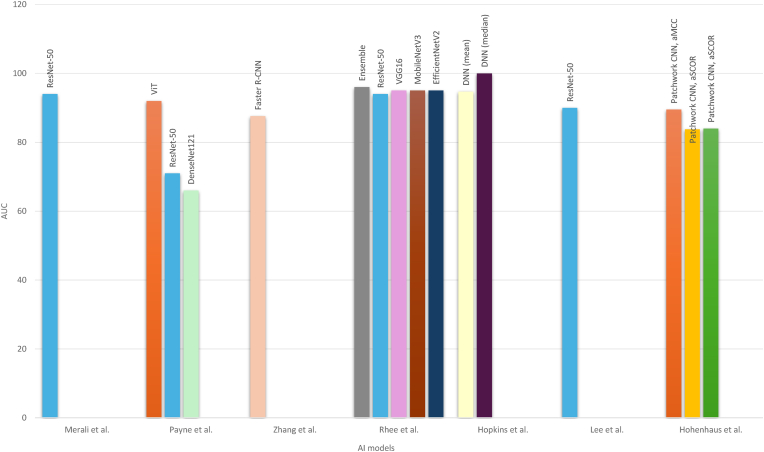
Comparison of AUC values across different AI models.

There exists substantial heterogeneity in the reporting of performance metrics of the AI models trained. These are presented in Table 6. AI model sensitivities across all of the included studies ranged from 0.67 to 1.0, with most models demonstrating values above 0.85. Accordingly specificities in the overwhelming majority of all AI models was located above 0.80 with just 1 model(Payne et al., 2024) demonstrating a specificity of 0.42. The accuracy of most models lied above 82 % supporting the robust performance of AI models in automated detection of cervical canal stenosis. The AUC in the majority of studies was reported above 0.90 indicating good generalization performance. A grouped bar chart comparing AUC values across studies is presented in Fig. 2. The reported k-value of the AI models showed substantial to near-perfect agreement with the labellers. The process time was reported only in 2 studies, both being ≤180 s, showing a significant reduction in the processing time for the diagnosis of cervical canal stenosis. Despite being less reported, F1-scores remained >80 % and recall rates >95 %. PPV and NPV metrics, when not reported, were calculated using the available confusion matrices and supplementary material. Finally, a pooled descriptive range analysis of the reported AUC and accuracy values is presented in Table 7. For studies that reported the performance of multiple AI models or applied different evaluation modalities, only the primary AI model or the highest-performing result was included to avoid data duplication.

**Table 6:** Performance metrics of AI models. (NR = not reported).

Author	Sensitivity	Sensitivity CI	Specificity	Specificity CI	AUC	AUC CI
Merali et al. (Merali et al., 2021)	0.88	NR	0.89	NR	0.94	NR
Payne et al. (Payne et al., 2024)	0.90 (ViT)	NR	0.95 (ViT)	NR	0.92 (ViT)	0.80–1.00(ViT)
	1.0 (ResNet-50)		0.42 (Resnet-50)		0.71 (ResNet-50)	
	0.8 (DenseNet121)		0.52 (DenseNet121)		0.66 (DenseNet121)	
Lee et al. (Lee et al., 2025a)	0.837 (internal)	0.7875–0.879 (internal)	0.9831 (internal)	0.9753–0.9889 (internal)	NR	NR
	0.9185 (external)	0.8999–0.937 (external)	0.9574 (external)	0.9496–0.9652 (external)		
Zhang et al. (Zhang et al., 2024)	0.835	NR	0.819	NR	0.876	0.865–0.886
Rhee et al. (Rhee et al., 2025)	0.885 (Ensemble model)	0.855–0.915 (Ensemble model)	0.861 (Ensemble model)	0.824–0.898 (Ensemble model)	0.96 (Ensemble model)	0.94–0.97 (Ensemble model)
	0.887 (ResNet-50)	0.857–0.917 (ResNet-50)	0.792 (ResNet-50)	0.748–0.835 (ResNet-50)	0.94 (ResNet-50)	0.93–0.96 (ResNet-50)
	0.906 (VGG16)	0.878–0.933 (VGG16)	0.843 (VGG16)	0.804–0.882 (VGG16)	0.95 (VGG16)	0.94–0.97 (VGG16)
	0.869 (MobileNetV3)	0.837–0.900 (MobileNetV3)	0.864 (MobileNetV3)	0.827–0.901 (MobileNetV3)	0.95 (MobileNetV3)	0.93–0.96 (MobileNetV3)
	0.866 (EfficientNetV2)	0.834–0.898 (EfficientNetV2)	0.870 (EfficientNetV2)	0.834–0.906 (EfficientNetV2)	0.95 (EfficientNetV2)	0.93–0.96 (EfficientNetV2)
Wang et al. (Wang et al., 2025)	0.884–0.90 (weighted, relative to labeller)	NR	0.951–0.961 (weighted, relative to labeller)		NR	NR
Hopkins et al. (Hopkins et al., 2019)	0.9025	NR	0.8505		0.947 (mean value)	NR
					1.0 (median value)	
Lee A. et al. (Lee et al., 2025b)	0.886	0.813–0.938	0.917	0.863–0.955	0.90	0.87–0.94
Abuhayi et al. (Abuhayi et al., 2024)	NR	NR	NR		NR	NR
Hohenhaus et al. (Hohenhaus et al., 2024)	0.78–0.81 (aMCC)	NR	0.80–0.82 (aMCC) 0.81–0.87 (aSCOR)		0.895 (aMCC)	NR
	0.67–0.78 (aSCOR)				0.838–0.840 (aSCOR)	

Author	PPV	NPV	Process Time (seconds)	Accuracy	k-value of AI to ground truth
Merali et al. (Merali et al., 2021)	NR	NR	NR	NR	NR
Payne et al. (Payne et al., 2024)	90 % (ViT)	95 % (ViT)	NR	82 % (at section-level)	NR
	47 % (ResNet-50)	100 % (ResNet-50)		93 % (at patient level) for ViT;	
	47 % (DenseNet121)	83 % (DenseNet121)		72 %(at section-level)	
				62 %(at patient level) for ResNet-50;	
				78 % (at section-level)	
				62 % (at patient level) for DenseNet121	
Lee et al. (Lee et al., 2025a)	89.68 % (internal)	97.17 % (internal)	NR	NR	0.78–0.95 (internal)
	87.44 % (internal)	97.32 % (external)			0.76–0.92 (external)
Zhang et al. (Zhang et al., 2024)	NR	NR	0.098 per slice	82.7 %	0.649
Rhee et al. (Rhee et al., 2025)	89.3 %(Ensemble model)	85.1 %(Ensemble model)	NR	87.5 % (Ensemble model)	NR
	84.8 %(ResNet-50)	84.2 %(ResNet-50)		84.6 % (ResNet-50)	
	88.3 %(VGG16)	87.2 %(VGG16)		87.8 % (VGG16)	
	89.3 %(MobileNetV3)	83.4 %(MobileNetV3)		86.7 % (MobileNetV3)	
	89.7 %(EfficientNetV2)	83.2 %(EfficientNetV2)		86.8 % (EfficientNetV2)	
Wang et al. (Wang et al., 2025)	NR	NR	NR	92.3 %–93.3 % (weighted, relative to labeller)	0.848 (vs. clinician 1)
					0.822 (vs. clinician 2)
					0.702 0.732 (vs. residents)
Hopkins et al. (Hopkins et al., 2019)	81.58 %	91.94 %	NR	86.50 % (mean value)	NR
				90.00 % (median value)	
Lee A. et al. (Lee et al., 2025b)	NR	NR	NR	NR	0.81
Abuhayi et al. (Abuhayi et al., 2024)	99.36 %	98.74 %	NR	98.73 %	NR
Hohenhaus et al. (Hohenhaus et al., 2024)	NR	NR	180	NR	NR

Author	Recall Rate	F1-Score
Merali et al. (Merali et al., 2021)	NR	0.82
Payne et al. (Payne et al., 2024)	NR	NR
Lee et al. (Lee et al., 2025a)	99.8 % (internal) 99.9 % (external)	NR
Zhang et al. (Zhang et al., 2024)	99.7 %	0.848
Rhee et al. (Rhee et al., 2025)	NR	NR
Wang et al. (Wang et al., 2025)	NR	0.884–0.901 (weighted, relative to labeller)
Hopkins et al. (Hopkins et al., 2019)	NR	NR
Lee A. et al. (Lee et al., 2025b)	NR	NR
Abuhayi et al. (Abuhayi et al., 2024)	NR	NR
Hohenhaus et al. (Hohenhaus et al., 2024)	NR	NR

**Table 7:** Pooled range analysis of AUC and accuracy values.

	Accuracy	AUC
Number of Studies (n)	6	7
Mean Accuracy	0.9028	0.9197
Median Accuracy	0.9025	0.920
Range	0.827–0.987	0.876–0.960
mean ± SD	0.0575	0.031

## 4 Discussion

This systematic review evaluated the diagnostic performance of artificial intelligence (AI) deep learning algorithms for the automated detection of cervical spinal stenosis (CCSS) on MRI. Across 10 included studies, AI models consistently demonstrated high sensitivity, specificity, and overall accuracy, with an AUC-Score across the overwhelming majority of the included studies exceeding 0.90. These findings suggest that AI-based approaches, particularly convolutional neural networks (CNN) and Transformer-based architectures, have strong potential to be utilized as automated tools for the diagnosis of cervical spinal stenosis. However, these performance estimates were largely derived from single-centre, retrospective cohorts and were frequently reported without confidence intervals, which limits precision and generalisability.

The results of the current systematic review align with other literature regarding AI use in spine imaging, where CNN-based models have achieved high diagnostic accuracy in tasks such as detection of lumbar degenerative disc disease, lumbar stenosis as well as improved productivity using AI-assisted reporting(Wang et al., 2024; Lim et al., 2022; Liawrungrueang et al., 2025b). While most prior systematic reviews have focused on the lumbar spine or broader degenerative changes, our analysis specifically targets cervical central spinal stenosis — a pathology with high clinical importance due to its risk of myelopathy — and its automated diagnosis. Importantly, the definition of “stenosis” varied across studies (e.g., Kang vs Muhle grading; binary vs ordinal dichotomisations), and this label heterogeneity likely contributes to between-study variation and complicates head-to-head comparisons.

The integration of AI-based automated detection tools into clinical workflows could improve efficiency, reduce inter-reader variability, and support earlier detection of clinically significant CCSS. These models may be particularly valuable in high-volume imaging centres and resource-limited settings. Moreover, standardized automated grading could enhance surgical decision-making and monitoring of disease progression. However, clinical deployment will require rigorous prospective validation, regulatory approval, and integration into radiology information systems. For clinical adoption, models must be well-calibrated (i.e., predicted probabilities reflect true risk), their thresholds pre-specified for actionable use cases (triage vs rule-out vs rule-in), and their impact demonstrated on workflow and patient-centred endpoints rather than image-only metrics.

## 5 Clinical implications and deployment

Across single-centre studies, high sensitivity and AUC suggest utility for prioritizing and standardizing reads in high-volume settings. Prospective studies should quantify end-to-end workflow impact—time-to-report, inter-reader variability, and concordance with surgical decisions—rather than image-only metrics. Integration will also depend on calibration, threshold selection for clinically actionable outputs, and robust handling of out-of-distribution cases (e.g., congenital stenosis, implants, motion artefact). Minimal-risk deployment pathways include decision support with human-in-the-loop review and audit trails. Finally, harmonization strategies (vendor/sequence normalization) and site-agnostic validation are prerequisites for scale. In practice, a staged pathway is pragmatic: (1) retrospective validation with subject-level splits and pre-specified thresholds; (2) silent prospective evaluation to monitor failure modes, calibration drift, and run-time; and (3) controlled deployment for triage or quality-assurance with clear escalation rules. Because CCSS prevalence is modest and class imbalance common, reliance on AUC alone can be misleading; reporting should include precision-recall curves, F1-scores with confidence intervals, and confusion-matrix metrics at the intended decision threshold. Generalisability depends on robust domain-shift defences—scanner vendor/coil/field-strength variation, protocol differences, and population mix. Techniques such as intensity harmonization, physics-aware augmentation, or ComBat-style normalization may help, but external, multi-centre testing remains the gold standard. Finally, explainability (e.g., Grad-CAM) should be paired with reader studies to verify that heatmaps highlight anatomically plausible regions and do not spuriously track artefacts or labels.

## 6 Limitations of the reviewed studies

The reviewed articles present a number of limitations. First, not all studies reported confidence intervals for sensitivity and specificity, limiting the assessment of statistical precision and comparability across models. Second, few studies employed or reported the use of explainability tools such as Grad-CAM, which are essential for understanding the decision-making process of deep learning algorithms and enhancing clinical interpretability. Third, there is a lack of consistency in reporting standards across studies, which complicates data synthesis and hinders the possibility of conducting robust meta-analyses. Adopting standardized reporting frameworks, such as CLAIM, would improve transparency and enable meaningful comparisons across studies. Other limitations of the included studies should be acknowledged. Most studies employed retrospective designs and single-centre datasets, which may limit generalisability. Sample sizes were variable, with some studies using relatively small test sets. External validation was performed in only 1 of the studies. Many datasets often came from similar populations or scanner types, limiting true heterogeneity assessment. Furthermore, reporting of performance metrics was inconsistent, with some studies omitting F1-scores, processing time, or detailed confidence intervals. Several studies split data at the image or slice level rather than at the patient level, creating a risk of information leakage and optimistic results. Thresholds were often tuned post hoc on test sets or not pre-specified, and model calibration was rarely reported—both hinder reproducibility and decision-focused evaluation. Label noise (inter-reader disagreement, differing grading systems) and conversion of ordinal grades to binary targets may inflate apparent performance; ordinal-aware losses or cumulative-link models could be more appropriate. Fairness and subgroup performance (e.g., sex/age, congenital vs acquired stenosis, OPLL, postoperative change) were not examined, leaving equity and failure-mode profiles unclear.

## 7 Limitations of this review

While this review followed PRISMA methodology, certain limitations apply. First, heterogeneity in study design, AI model architecture, and outcome reporting precluded quantitative meta-analysis. Second, we excluded non-MRI studies and AI models applied to segmentation or enhancement, which may indirectly inform diagnostic models. Publication bias is possible, as studies with poor performance may be underrepresented in the literature. Finally, we performed a narrative risk-of-bias appraisal (aligned to AI-specific domains such as leakage, thresholding, and external validation) rather than a formal diagnostic-test instrument, because reporting was insufficiently consistent to score studies reliably.

## 8 Future directions

Priorities include large-scale, multi-centre, prospective studies with standardized MRI protocols and transparent AI reporting; external validation across diverse scanners, institutions, and demographics; and consistent explainability reporting. Prospective pragmatic designs—before/after or stepped-wedge cluster trials—should quantify effects on workflow and clinical decisions, with randomised evaluations considered for high-stakes automation. Protocol pre-registration, pre-specified thresholds, patient-level splitting, and full confidence intervals should be standard. Studies should report calibration, uncertainty, and subgroup performance, and release code and (where possible) benchmarking datasets with standardized CCSS grading. Combining imaging with structured clinical variables (e.g., mJOA) may yield models that better map to actionable decisions.

## 9 Conclusions

AI deep learning models show strong potential for accurately detecting cervical spinal stenosis on MRI. While diagnostic performance is consistently high, standardisation, external validation, calibration/threshold reporting, and prospective workflow evaluation are needed before widespread clinical adoption.

## Ethics approval

Not applicable (systematic review).

## Funding

None declared.

## Declaration of competing interest

The authors declare that they have no known competing financial interests or personal relationships that could have appeared to influence the work reported in this paper.

## Data Availability

All data are extracted from published articles cited herein.
