# Supplementary material for: Application and performance of deep learning models for the automated diagnosis of cervical central spinal stenosis on MRI: a systematic review
Source: Brain Spine. 2025 Dec 18;6:105902. doi: 10.1016/j.bas.2025.105902 (PMC12796762; doi:10.1016/j.bas.2025.105902)
Supplement: Multimedia component 1 [file mmc1.docx]

**Supplementary Table 1.** Database search strings

| PubMed | ((( "cervical spinal stenosis"[Title/Abstract] OR "cervical canal stenosis"[Title/Abstract] OR "cervical spine"[Title/Abstract] OR "spinal stenosis"[MeSH Terms] ) AND ( "magnetic resonance imaging"[MeSH Terms] OR MRI[Title/Abstract] OR "MR imaging"[Title/Abstract] ) AND ( "artificial intelligence"[MeSH Terms] OR AI[Title/Abstract] OR "machine learning"[Title/Abstract] OR "deep learning"[Title/Abstract] OR "neural network"[Title/Abstract] OR "convolutional neural network"[Title/Abstract] OR CNN[Title/Abstract] )) OR (( "cervical"[Title/Abstract] OR "cervical spine"[Title/Abstract] OR "cervical vertebrae"[Title/Abstract] OR "cervical spinal stenosis"[Title/Abstract] OR "cervical canal stenosis"[Title/Abstract] OR "cervical stenosis"[Title/Abstract] OR "spinal stenosis"[Title/Abstract] OR "spinal canal narrowing"[Title/Abstract] OR "spinal compression"[Title/Abstract] OR "spine diseases"[MeSH Terms] ) AND ( "magnetic resonance imaging"[MeSH Terms] OR "MRI"[Title/Abstract] OR "MR imaging"[Title/Abstract] OR "magnetic resonance"[Title/Abstract] OR "neuroimaging"[Title/Abstract] OR "spinal imaging"[Title/Abstract] ) AND ( "artificial intelligence"[MeSH Terms] OR "machine learning"[MeSH Terms] OR "deep learning"[Title/Abstract] OR "AI"[Title/Abstract] OR "machine learning"[Title/Abstract] OR "neural network"[Title/Abstract] OR "deep neural network"[Title/Abstract] OR "convolutional neural network"[Title/Abstract] OR "CNN"[Title/Abstract] OR "computer vision"[Title/Abstract] OR "automated diagnosis"[Title/Abstract] OR "image analysis"[Title/Abstract] OR "CAD"[Title/Abstract] OR "pattern recognition"[Title/Abstract] ))) NOT ( "lumbar"[Title/Abstract] OR "lumbar spine"[Title/Abstract] OR "lumbar spinal stenosis"[Title/Abstract] ) AND ("2015/01/01"[Date - Publication] : "2025/07/31"[Date - Publication]) |
| --- | --- |
| Cochrane | ("artificial intelligence":ti,ab OR "machine learning":ti,ab OR "deep learning":ti,ab OR "neural network*":ti,ab OR "computer-assisted diagnosis":ti,ab OR AI:ti,ab OR CNN:ti,ab OR "convolutional neural network*":ti,ab) AND ("magnetic resonance imaging":ti,ab OR MRI:ti,ab) AND ("cervical spinal stenosis":ti,ab OR "cervical canal stenosis":ti,ab OR ("cervical stenosis":ti,ab AND spine:ti,ab)) NOT ("cervical cancer":ti,ab OR "cervical dysplasia":ti,ab OR "cervical lymph nodes":ti,ab OR "cervical carcinoma":ti,ab OR prostate:ti,ab OR thoracic:ti,ab OR lumbar:ti,ab OR sacral:ti,ab OR pelvic:ti,ab OR uterus:ti,ab) |
| EMBASE | ('cervical spine'/exp OR 'cervical vertebra'/exp OR 'cervical spinal stenosis':ti,ab OR 'cervical canal stenosis':ti,ab OR 'cervical stenosis':ti,ab OR ('cervical':ti,ab AND ('spine':ti,ab OR 'vertebrae':ti,ab OR 'canal narrowing':ti,ab OR 'compression':ti,ab OR 'stenosis':ti,ab)) OR 'spinal stenosis'/exp OR 'spinal canal stenosis':ti,ab) AND ('magnetic resonance imaging'/exp OR 'mri':ti,ab OR 'mr imaging':ti,ab OR 'magnetic resonance':ti,ab OR 'spinal imaging':ti,ab OR 'neuroimaging':ti,ab) AND ('artificial intelligence'/exp OR 'machine learning'/exp OR 'deep learning':ti,ab OR 'ai':ti,ab OR 'neural network':ti,ab OR 'convolutional neural network':ti,ab OR 'cnn':ti,ab OR 'computer vision':ti,ab OR 'automated diagnosis':ti,ab OR 'image analysis':ti,ab OR 'pattern recognition':ti,ab OR 'cad':ti,ab) NOT ('lumbar spine'/exp OR 'lumbar spinal stenosis':ti,ab OR 'thoracic spine'/exp) AND [2015-2025]/py AND [humans]/lim |
| IEEEXPLORE | ( ("cervical" OR "cervical canal stenosis" OR "cervical spinal stenosis" OR "spinal compression") ) AND ( ("MRI" OR "magnetic resonance" OR "spinal imaging" OR "medical imaging") ) AND ( ("machine learning" OR "deep learning" OR "convolutional neural network" OR "CNN") ) |
| Web of Science | TS=("cervical spine" OR "cervical vertebra" OR "neck vertebra" OR "cervical spinal stenosis" OR "cervical canal stenosis" OR "cervical stenosis" OR "cervical spondylosis" OR "cervical myelopathy") AND TS=("magnetic resonance imaging" OR "mri" OR "mr imaging" OR "magnetic resonance" OR "spinal imaging" OR "neuroimaging") AND TS=("artificial intelligence" OR "machine learning" OR "deep learning" OR "ai" OR "neural network" OR "cnn" OR "computer vision" OR "automated diagnosis") NOT TS=("lumbar spine" OR "thoracic spine")  OR  ("cervical spine" OR "cervical vertebra" OR "cervical spinal stenosis" OR "cervical canal stenosis" OR "cervical stenosis" OR "spinal stenosis" OR "spinal canal stenosis" OR cervical spine OR cervical vertebrae OR cervical "canal narrowing" OR cervical compression OR cervical stenosis) AND ("magnetic resonance imaging" OR mri OR "mr imaging" OR "magnetic resonance" OR "spinal imaging" OR neuroimaging) AND ("artificial intelligence" OR "machine learning" OR "deep learning" OR ai OR "neural network" OR "convolutional neural network" OR cnn OR "computer vision" OR "automated diagnosis" OR "image analysis" OR "pattern recognition" OR cad) NOT ("lumbar spine" OR "lumbar spinal stenosis" OR "thoracic spine") (All Fields) |
